# Current status of therapeutic approaches and vaccines for SARS-CoV-2

**DOI:** 10.2217/fmb-2020-0147

**Published:** 2021-11-11

**Authors:** Braira Wahid, Anam Amir, Ayesha Ameen, Muhammad Idrees

**Affiliations:** ^1^Monash Biomedicine Discovery Institute, Department of Microbiology, Monash University, Clayton, Australia; ^2^Department of Life Sciences, School of Sciences, University of Management & Technology, Lahore, Pakistan; ^3^Office of Research, Innovation, & Commercialization, University of Management & Technology, Lahore, Pakistan; ^4^Centre of Excellence in Molecular Biology, University of The Punjab, Lahore, Pakistan

**Keywords:** antivirals, chloroquine, COVID-2019, epidemic, pandemic, SARS-CoV-2, therapeutics, treatment, vaccines

## Abstract

SARS-CoV-2, declared a pandemic in March 2020, is the current global health challenge. The global bioburden of this virus is increasing at a rapid pace. Many antiviral drugs and vaccines have been registered for clinical trials because of their inhibitory activity observed *in vitro*. Currently, five types of vaccines have successfully passed Phase IV clinical trial and are being administered in populations worldwide. A plethora of experimental designs have been proposed worldwide in order to find a safe and efficacious treatment option. Therefore, it is necessary to provide baseline data and information to clinicians and researchers so that they can review the current status of therapeutics and efficacy of already developed vaccines. This review article summarizes all therapeutic options that may help to combat SARS-CoV-2.

COVID-19, caused by SARS-CoV-2, is a global health emergency that was first reported in Wuhan, China, in December 2019. Initially, it was a regional epidemic, but it was later declared a pandemic in March 2020, due to its transmission to almost all countries of the world. As of 28 September 2021, 219 million people has been infected with the virus, and 4.55 million have died of it [1].

SARS-CoV-2 is positive-sense RNA virus that belongs to the family Coronaviridae*.* Coronaviruses are further classified into four genera known as α, β, γ and δ. α and β coronaviruses are reported to infect humans, and γ and δ coronaviruses infect birds. The other two major types of β-coronaviruses are SARS-CoV and MERS-CoV, which caused outbreaks in China in 2003 and in Saudi Arabia in 2012. The genome sequences of SARS-CoV, MERS-CoV and SARS-CoV-2 are similar, but the SARS-CoV-2 genome is more closely related to MERS-CoV than SARS-CoV [2,3].

Clinical manifestation of SARS-CoV-2 varies from mild symptoms such as fever and flu to severe symptoms with respiratory complications, septic shock, multiple organ failure and cardiogenic shock [4]. The most common mode of transmission is respiratory droplets of infected persons that spread in the surrounding environment and on surfaces during coughing and sneezing. However, some evidence has also suggested its transmission through the oral–fecal route; therefore, further research is needed to explore other routes of transmission of the virus [5].

At the time of writing, there is no specific treatment available for SARS-CoV-2, but scientists are continually seeking to develop vaccines and effective drugs to treat SARS-CoV-2 infection. More than 30 drugs and seven types of vaccines are at the time of writing under trial. Some research groups are studying the therapeutic potential of plasma isolated from recovered patients [6]. This review briefly describes the current status of SARS-CoV-2 therapeutics and provides ample information to the research community to understand the anticipated therapeutic potential of various therapeutic approaches that are, at the time of writing, under clinical trial (Table 1).

**Table 1.** Medicinal products for SARS-CoV-2.

Medicinal product	Current status of research
**Vaccines**	
RNA	LNP-encapsulated mRNA (Phase III clinical trial) Moderna (Phase IV clinical trial)/National Institute of Allergy and Infectious Diseases
	Three LNP-mRNAs (Phase III clinical trial) BioNTech/Fosun Pharma/Pfizer (Phase IV clinical trial)
DNA	DNA plasmid vaccine with electroporation (Phase I/II clinical trial) Inovio Pharmaceuticals/International Vaccine Institute
	DNA plasmid vaccine + Adjuvant (Phase III clinical trial) Osaka University/AnGes/Takara Bio
	DNA plasmid vaccine (Phase I/II clinical trial) Cadila Healthcare Limited
	DNA Vaccine (GX-19) (Phase I/II clinical trial) Genexine Consortium
Inactivated	Sinovac (Phase IV clinical trial) Sinopharm (Phase IV clinical trial) Bharat Biotech (Phase III clinical trial)
Nonreplicating viral vector	ChAdOx1-S (Phase III clinical trial) AstraZeneca (passed Phase IV clinical trial)
	Adenovirus type 5 Vector (Phase III clinical trial) Beijing Institute of Biotechnology
	Sputnik V Adeno-based (rAd26-S + rAd5-S) (Phase IV clinical trial) Gamaleya Research Institute
	Adenovirus type 26 vector (Phase III clinical trial) Janssen Pharmaceutical Companies
Protein subunit	Full length recombinant SARS CoV-2 glycoprotein nanoparticle vaccine adjuvanted with Matrix M (Phase III clinical trial) Novavax
100+ other vaccine candidates	Under preclinical evaluation (WHO)
**Antiviral drugs**	
Lopinavir with ritonavir	Entered clinical trials (WHO)
Lopinavir with ritonavir plus Interferon β-1a	Entered clinical trials (WHO)
Ivermectin	Entered clinical trials (WHO)
Remdesivir	Entered clinical trials (WHO)
**Immunotherapeutics**	
Convalescent therapy	Clinical trials (UK government, University of California – San Francisco, Turkish Health Ministry, Drug Regulatory Authority of Pakistan
Monoclonal antibody	Tocilizumab – clinical trials (National Institutes of Health – US National Library of Medicine)

## Vaccines

The previous efforts made for the development of SARS-CoV vaccine will assist scientists in developing vaccines against SARS-CoV-2 because of genetic similarities between both viruses and their capability to bind with the same host cell receptor, ACE2. Vaccine technologies that are under evaluation are nucleic acid vaccines, subunit vaccines, whole-virus vaccines and recombinant proteins [7].

An *in silico* study reported that B- and T-cell epitopes present in structural proteins of SARS-CoV-2 were identical to B- and T-cell epitopes present in nucleocapsid (N) and spike (S) protein of SARS-CoV-2. The identical epitopes were classified as discontinuous B-cell epitopes or conformational epitopes and linear B-cell epitopes or antigenic peptides. The study also highlighted that N and S regions of SARS-CoV-2 genome do not contain any mutation after analyzing 120 available sequences. Therefore, this conserved region (N and S) can help in the successful development of vaccines, which will offer protection by targeting these epitopes [8].

Another *in silico* study used bioinformatics tools to develop a multiepitope vaccine that can trigger the CD4+ and CD8+ T-cell-based immune responses. A set of three antigenic proteins known as NOM (N protein, open reading frame 3a and membrane protein) were analyzed in the study to predict the potential of B- and T-cell epitopes. NOM-based multiepitope vaccine with five rich epitopes was constructed, followed by molecular docking with HLA-A*11:01 and Toll-like receptor 4 (TLR4). The study also included molecular dynamics simulation to monitor the stability of NOM recombinant vaccine candidates with HLA-A*11:01 and TLR4 [9].

Moderna, a UK-based company, and China’s CanSino Biologics were the first two companies to launch clinical trials for SARS-CoV-2 vaccines. Different vaccine candidates that have entered Phase IV clinical trials are adenovirus type 5 vector (CanSino Biological Inc., Beijing, China), ChAdOx1 (University of Oxford, Oxford, UK), DNA plasmid vaccine Electroporation device (Inovio Pharmaceuticals, PA, USA), Inactivated (Beijing Institute of Biological Products, Beijing, China), inactivated + alum (Sinovac Biotech Ltd, Beijing, China), mRNA (by BioNTech, Germany/Pfizer, NY, USA/Fosun Pharma, Shanghai, China) and LNP-encapsulated mRNA (National Institute of Allergy and Infectious Diseases, ML, USA/Moderna, MA, USA) [10]. The DNA–plasmid approach is a genetically engineered technique used by Inovio Pharmaceuticals. This company has used the same approach against MERS-CoV infection. The development of DNA-based plasmid vaccine depends on the transfer of a genetic blueprint of RNA present in the host cell machinery, which makes spike antigens. Some researchers directly embedded blueprint in an mRNA strand instead of using plasmid to develop an mRNA vaccine. RNA is infused in the body of a person with the help of lipid carriers that can easily pass into cells due to the presence of fatty molecules during the development of an mRNA vaccine. According to some researchers, RNA vaccines will be more efficacious than DNA plasmid vaccines in triggering the immune system to generate antibodies and may induce more potent and long-term immunity with low dose frequency.

Sinovac Biotech was the first to carry out a vaccine trial on animal models. Eight rhesus macaques were included in the study and administered two doses of a SARS-CoV-2 vaccine. The animals were later exposed to SARS-CoV-2, but none showed any symptoms of infection. On the basis of the positive therapeutic outcome observed in monkeys, the company has taken the vaccine to human trials.

The WHO harnessed global cooperation among public and private partners to develop a vaccine as soon as possible. The WHO has established a platform to follow the progress of all vaccines that have been proposed worldwide. There are, at the time of writing, 184 vaccines in preclinical evaluation, and 104 vaccines have entered clinical evaluation. Six vaccine candidates (Pfizer, Moderna, Sputnik V, Sinovac, Sinopharm and AstraZeneca) are in Phase IV clinical trials and administered to populations.

## Antiviral drugs

An antiviral drug against SARS-CoV-2 has not yet been developed. However, many drugs are under preclinical and clinical evaluation. Antiviral therapies that are, at the time of writing, in clinical trials can be classified into two major categories depending on the target. Some antiviral drugs are meant to control SARS-CoV-2 either by blocking entry of virus in cells or by inhibition of gene replication enzyme. The other type of drugs will target the host by triggering the innate immune response of an infected person. Most of the host-targeted antiviral drugs were designed for previously reported coronaviruses, and now the same drugs are reanalyzed to treat SARS-CoV-2. An *in silico* study based on 120 broad-spectrum antiviral agents checked the efficacy of these drugs against SARS-CoV-2 and found out that emetine, monensin, dalbavancin, oritavancin and teicoplanin could be effective against SARS-CoV-2. Moreover, several randomized clinical trials have been registered to test the efficacy of chloroquine, ritonavir, arabido, lopinavir and remdesivir due to their proven ability to inhibit SARS-CoV-2 infection *in vitro* [11]. Gordon *et al.* studied 332 SARS-CoV-2 human protein–protein interactions, of which 66 could be targeted by US FDA-approved drugs. Scientists are now analyzing the efficacy of these drugs *in vitro* [12].

Likewise, another study reported that the genetic sequence of SARS-CoV-2 is 96% similar to other coronaviruses. Protease and spike proteins of SARS-CoV-2 have 96 and 75% genetic similarity to the protease and spike proteins of other coronaviruses, such as MERS and SARS-CoV [13,14]. The study proposed that an effective SARS-CoV-2 drug can be easily developed by targeting protease and spike proteins because previous experiments confirmed the role of some inhibitors of protease and spike protein in the control of SARS and MERS virus [15–17]. This study also involved the molecular docking of 13 antiviral agents being used for treatment of HIV and HCV to assess the affinity of these antiviral agents with SARS-CoV-2. Of 13 protease inhibitors only enfuvirtide, umifenovir and pleconaril had a high affinity with the drug in thermodynamic terms [18]. Another study has also shown potential drug targets such as main protease, Nsp12 RNA polymerase and Nsp13 helicase of SARS-CoV-2 [19]. Dai *et al.* reported that two compounds, 11a and 11b, are potent inhibitors of main protease enzyme of SARS-CoV-2, which plays an important role in virus replication and transcription [20].

Findings of some experiments have confirmed remdesivir (a nucleotide analogue of RNA-dependent RNA polymerase) as a promising antiviral agent to control SARS-CoV-2. The structural and functional properties of remdesivir (GS-5734, Gilead Sciences, CA, USA) were similar to tenofovir (a hepatitis B drug and nucleotide analog of adenosine 5-monophosphate) [21]. Remdesivir has also shown antiviral activity in an experiment based on the use of Vero E6 cells. In addition, remdesivir had been found to be effective against Nipah virus, Marburg virus, measles and mumps virus, Hendra virus, parainfluenza type 3 virus, Ebola virus and respiratory syncytial virus [22–24]. Remdesivir has also been found to be effective against zoonotic coronaviruses such as mouse hepatitis virus, HCoV-OC43, HCoV-NL63, HCoV-229E, SARS-CoV, MERS-CoV, porcine deltacoronavirus and pipistrellus bat coronavirus *HKU5*. In another study, remdesivir inhibited the replication of MERS-CoV (half maximal inhibitory concentration, IC_50_: 0.074 μM) and SARS-CoV (IC_50_: 0.069 μM) when tested on primary human airway epithelial cell culture [21,25,26].

Gilead Sciences conducted a study to test the efficacy of remdesivir and recruited two groups of six rhesus macaques; one group was untreated and the macaques of the other group were treated with remdesivir. Twelve hours after receiving the first dose of treatment, improvement in health was observed and after several doses, and viral load was lower in the lungs of treated participants compared with untreated participants [27].

The first successful case of treatment of SARS-CoV-2 with remdesivir was reported in WA, USA. Remdesivir was intravenously administered to a SARS-CoV-2 positive patient who had also developed pneumonia. In response to remdesivir, the patient showed signs of improvement with no side effects. However, detection of SARS-CoV-2 by processing nasopharyngeal swab in reverse transcriptase PCR displayed positive results even after a 4-day treatment with remdesivir, but authors observed a constant decrease in viral load during treatment duration [28]. Remdesivir has entered Phase III clinical trials in China to test its efficacy and safety profile following a treatment regimen and pattern that had already been used in a randomized clinical trials with the Ebola virus [29,30]. Approximately 237 SARS-CoV-2-positive patients with symptoms of pneumonia were recruited in a randomized placebo-controlled clinical trial. Intravenous remdesivir (200 mg on day 1 and 100 mg for the next 9 days) and placebo infusions were administered to patients for 10 days. Recruited participants were also allowed to use corticosteroids, interferons and lopinavir–ritonavir. The clinical outcome of the drug was monitored regularly, and 18 patients from remdesivir-treated group and five from the placebo group experience adverse events. The results indicated that remdesivir is not an effective antiviral agent and does not play any role in accelerating SARS-CoV-2 treatment [31]. In contrast, another study reported that 68% of patients (36 of 53) showed signs of recovery with compassionate use of remdesivir [32]. Likewise, another study has proposed the mechanism of action of remdesivir and found that viral polymerase and proofreading exribonuclease mediates susceptibility of coronavirus to remdesivir. The development of antiviral nucleoside coronaviruses has been complicated due to the presence of CoV nsp14 exoribonuclease (ExoN). Although many of the studies showed that remdesivir has the ability to inhibit coronaviruses *in vitro*, in both animal models and human subjects, only one study has addressed the resistance associated with remdesivir and demonstrated that viral mutants that lack ExoN proofreading activity were more sensitive to remdesivir. Further analysis showed that remdesivir targets nsp12 polymerase, even when ExoN proofreading activity is intact and resistance can be easily overcome by increasing the dose concentration and frequency of remdesivir. Although accumulating evidence has proven that remdesivir is a broad-spectrum antiviral agent, according to recent studies, it is not potent against SARS-CoV-2; however, further studies are needed to confirm the efficacy of remdesivir for treatment of SARS-CoV-2 [21,33,34]. Another clinical trial known as RECOVERY, showed dexamethasone as a potent antiviral agent against SARS-CoV-2 [35].

Some of the studies have reported that use of chloroquine and its derivative hydroxychloroquine, accelerates the recovery time of SARS-CoV-2-infected patients and some evidence, based on *in vitro* studies, has suggested that the combination of remdesivir and chloroquine can more effectively inhibit SARS-CoV-2 infection [36]. Chloroquine has shown efficacy in Chinese patients infected with SARS-CoV-2. Further studies found that hydroxychloroquine is more safe and effective than chloroquine. Another experiment recruited two groups of patients (the first received hydroxychloroquine, and the second was treated with a combination of hydroxychloroquine and azithromycin). The findings indicated a rapid decrease in viral load in patients treated with a combination of hydroxychloroquine and azithromycin, compared with those treated with hydroxychloroquine only. Patients receiving monotherapy displayed respiratory complications [37–40]. In contrast to the previously reported findings of Gautret *et al.*, the latest findings suggest that chloroquinoline is not effective against SARS-CoV-2 [41]. However, the WHO has launched a Solidarity Clinical Trial to test the efficacy of different drugs based on their *in vitro* inhibitory effects against SARS-CoV-2. The drugs registered in this this trial are remdesivir; chloroquine; hydroxychloroquine; a combination of ritonavir and lopinavir; and a combination of ritonavir, interferon and lopinavir [42,43].

## Immunotherapeutic strategies

Different experiments have analyzed the effects of different immunotherapeutic approaches against SARS-CoV-2. Convalescent therapy is a classic immunotherapeutic method that has been used in the control of different infectious diseases over the past century. The safety and efficacy profile of convalescent therapy was found to be efficacious when used for the treatment of SARS-CoV and MERS [44–48].

A recently published study recruited ten SARS-CoV-2 positive patients who developed severe symptoms of infection. All participants were administered with 200 ml of convalescent plasma (CP) isolated from recovered patients with neutralizing antibodies. The clinical symptoms and viral load were periodically monitored after every dose. Patients showed significant improvement in the severity of disease symptoms with CP transfusions. They also showed an increase in oxyhemoglobin saturation and lymphocyte counts and decrease in C-reactive protein. The study confirmed that CP is a well-tolerated, safe and efficacious treatment option for patients infected with SARS-CoV-2 and may improve clinical outcome due to the presence of neutralizing antibodies in CP [6]. A similar case series has also been conducted in Shenzhen and confirmed the efficacy of convalescent plasma transfusion in five critically ill SARS-CoV-2 patients suffering from respiratory distress syndrome (ARDS). Clinical outcomes monitored before and after CP transfusion were compared in order to analyze the effect of neutralizing antibodies on SARS-CoV-2 viral load and other symptoms. The results showed that all patients were on mechanical ventilation before treatment, but after few transfusions their body temperature and viral load decreased, Pao2/Fio2 increased, and SOFA score decreased. After 2 weeks, patients were weaned off of mechanical ventilation and were discharged after 50 days in the hospital, during which time they did not develop complications [49]. The UK government; University of California, San Francisco; Turkish Health Ministry and the Drug Regulatory Authority of Pakistan have also approved the clinical trials of plasma therapy for SARS-CoV-2.

A monoclonal antibody, tocilizumab (TCZ), acts against the IL-6 receptor (IL-6R) and has been found effective in SARS-CoV-2 patients. IL-6, which is a cytokine, has been reported to cause an inflammatory reaction in SARS-CoV-2 patients. Cytokine storm, or cytokine release syndrome (CRS), induced by SARS-CoV-2 leads to cardiovascular collapse, acute respiratory distress syndrome, multiple organ failure and death, whereas elevation in other pro-inflammatory cytokines cause macrophage activation syndrome in SARS-CoV-2 patients. A retrospective study published by Luo *et al.* analyzed the efficacy of TCZ in 15 SARS-CoV-2 patients, of whom seven were critically ill, six were severely ill and two had mild symptoms. TCZ was administered with methylprednisolone twice a day. Ten patients showed a decrease in IL-6 level, but critically ill patients did not respond; persistent and dramatic IL-6 elevation was noted in these patients [50].

## Future perspective

Researchers worldwide are struggling with therapeutic challenges associated with SARS-CoV-2. Almost six vaccine candidates are in Phase IV clinical trials and are being administered to populations on a massive scale. Further research is required to analyze the efficacy and safety of vaccines and antiviral potential of lopinavir/ritonavir remdesivir, dexamethasone and umifenovir. Clinical manifestation of SARS-CoV-2 in the form of cytokine storm also highlights the need to conduct research on mesenchymal stem cells because of their anti-inflammatory and immune modulatory role.

## Conclusion

SARS-CoV-2 is a major global health challenge associated with 62.1 million affected and 1.45 million deaths worldwide as of this writing. SARS-CoV-2 originated in Wuhan, China, in December 2019 and was declared a pandemic in March 2020. The clinical severity of SARS-CoV-2, its presence in respiratory droplets in the air and the absence of preexisting immunity due to the novel nature of the virus have made all people susceptible. At the time of writing, no licensed therapy is available to treat SARS-CoV-2 infection; however, many drugs (remdesivir, chloroquinoline, lopinovir, ritonavir), vaccines (DNA, RNA, mRNA, inactivated, nonreplicated viral vector) and immunotherapeutic strategies (tocilizumab, siltuximab, adalimumab, plasma therapy) have entered clinical trials because of their inhibitory effects in human subjects and *in vitro*.

**Executive summary**

**Introduction**

- SARS-CoV-2 is the current global health challenge that was declared pandemic in March 2020.
- As of 28 September 2021, 219 million people had been infected with the virus, and 4.55 million had died of the infection.
- This review briefly describes the current status of SARS-CoV-2 therapeutics and provides ample information to researcher community to understand anticipated therapeutic potential of different therapeutic approaches that are, at the time of writing, in clinical trials.

**Vaccines**

- There are, at the time of writing, 184 vaccines in preclinical evaluation, and 104 vaccines have entered clinical evaluation.
- Almost six vaccine candidates (Pfizer, Moderna, Sputnik V, Sinovac, Sinopharm and AstraZeneca) are in Phase IV clinical trial and administered to populations at massive level.

**Antiviral drugs**

- An antiviral drug against SARS-CoV-2 has not yet been developed.
- However, many drugs are under preclinical and clinical evaluation.
- Antiviral therapies that are, at the time of writing, in clinical trials can be classified into two major categories depending on the target.
- Some antiviral drugs are meant to control SARS-CoV-2 either by blocking entry of virus in cells or by inhibition of gene replication enzyme.
- The other type of drug will target the host immunity by triggering the innate response in an infected person.
- The first successful case of treatment of SARS-CoV-2 with remdesivir was reported in WA, USA.

**Immunotherapeutic strategies**

- Experiments have analyzed the effects of different immunotherapeutic approaches against SARS-CoV-2.
- Convalescent therapy (or plasma therapy) has been found to be safe and effective among patients infected with SARS-CoV-2.
- The monoclonal antibody tocilizumab (TCZ), which acts against the IL-6 receptor, has shown efficacy in SARS-CoV-2 patients.

**Future perspective**

- Further research is required to analyze the efficacy and safety of vaccines and the antiviral potential of lopinavir/ritonavir remdesivir, dexamethasone and umifenovir.
